# An Atypical Manifestation: Linear Hyperkeratotic Prurigo Nodularis

**DOI:** 10.7759/cureus.74588

**Published:** 2024-11-27

**Authors:** Sanjana Likki, Iman Ali, T. Austin Black, Noel X Yang, Emelie N McQuitty, Rashid M Rashid

**Affiliations:** 1 Dermatology, University of Texas Health Science Center at Houston, John P. and Katherine G. McGovern Medical School, Houston, USA; 2 Dermatology, Mosaic Dermatology, Houston, USA

**Keywords:** dupilumab, hyperkeratosis, pruigo nodularis, prurigo, pruritus, skin nodules

## Abstract

We present the case of a 42-year-old Indian male with prurigo nodularis, exhibiting multiple verrucous, brownish-black nodules on the left lower and right upper extremities, with milder involvement of the right lower extremity. The lesions were asymmetrically distributed in a near-linear pattern, with prominent white hyperkeratosis and associated moderate pruritus and paresthesia. Differential diagnoses included pemphigoid nodularis, verruca vulgaris, and hypertrophic lichen planus, with a biopsy confirming prurigo nodularis. While prurigo nodularis typically presents with symmetric dome-shaped nodules on extensor surfaces, the asymmetric distribution, near-linear arrangement, and verrucous texture observed here are unusual and underscore the importance of recognizing prurigo nodularis variants to ensure accurate diagnosis and effective management.

## Introduction

Prurigo nodularis is characterized by multiple, isolated papules or nodules that often lead to intense and persistent itch [1]. Clinically, prurigo nodules present as dome-shaped, ulcerated, and excoriated lesions [2]. These nodules typically appear symmetrically on the extensor surfaces of the upper and lower extremities and range in color from flesh-toned to red, pink, black, or brown [2,3]. As the exact pathogenesis remains unknown, diagnosis is predominantly clinical, though the onset is occasionally linked to underlying conditions [1,2]. Given the chronic and intensely pruritic nature of prurigo nodularis, the disease significantly impacts patients’ quality of life, often contributing to high rates of anxiety and depression [3]. Suboptimal management further exacerbates these challenges, highlighting the critical need for standardized diagnostic algorithms to enhance the evaluation and treatment of this burdensome condition. Here, we present a novel case of verrucous prurigo nodularis with an unusual asymmetric, near-linear distribution on the bilateral lower and right upper extremities, accompanied by prominent white hyperkeratosis.

## Case presentation

A 42-year-old Indian male presented to the dermatology clinic with multiple verrucous, brownish-black nodules on the distal extremities. The patient first noticed lesions on his lower extremities approximately five years prior, which gradually spread to include the right upper extremity. During this period, the nodules increased in size and number, with new lesions appearing every few months. The patient experienced intermittent episodes of intense pruritus, sometimes causing bleeding from scratching. He denied any significant past medical history, family history of skin conditions, or relevant comorbidities.

**Figure 1:**
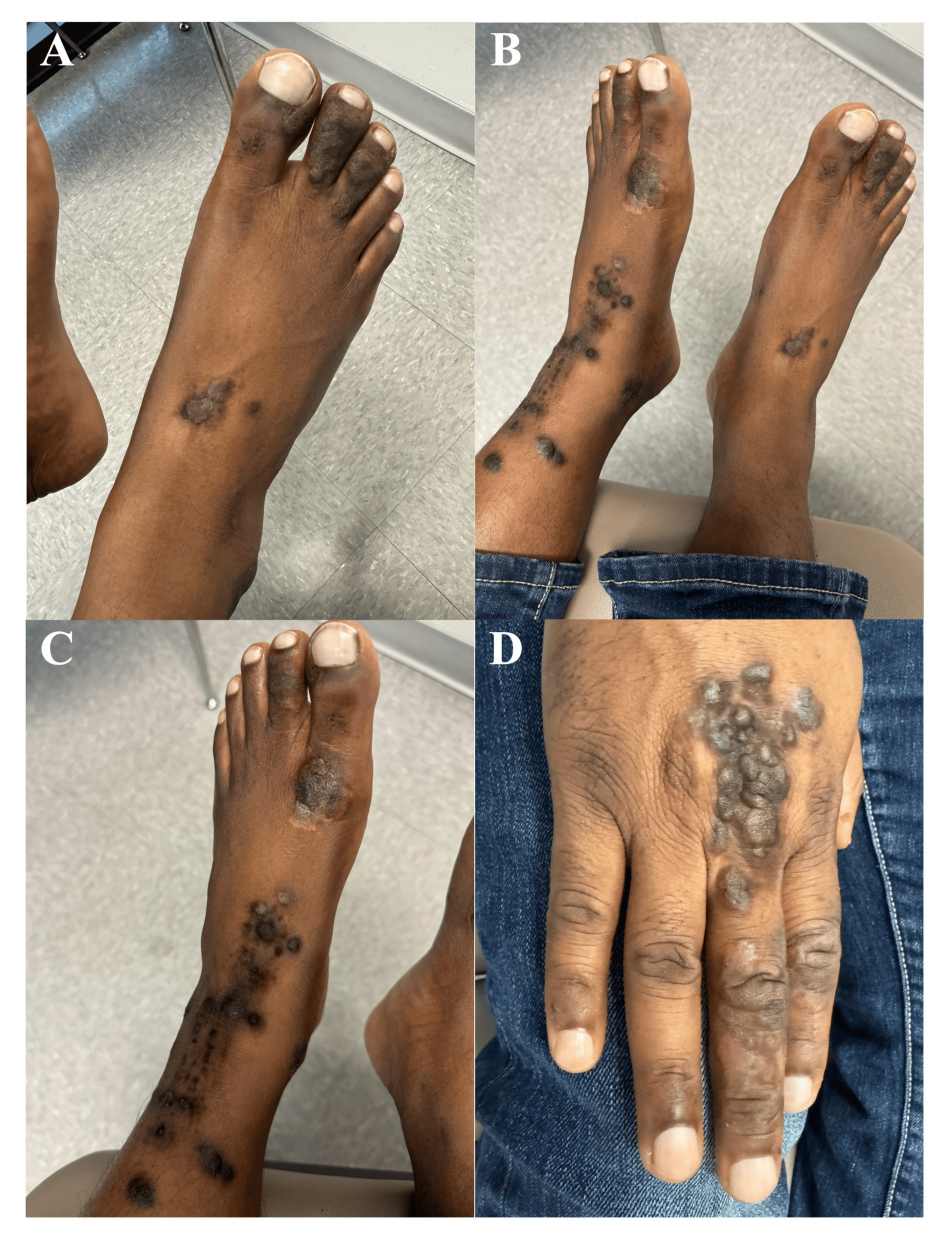
Prurigo nodules on the patient’s right foot (A) and left foot, extending to the anterior leg in a somewhat linear pattern (B). An additional view of the left lower extremity (C). Nodules on the right hand began proximally as a clustered pattern before transitioning to a more linear presentation on the third digit (D).

On physical examination, the lesions were asymmetrically distributed, predominantly on the left lower and right upper extremities, with less severe involvement of the right lower extremity. Nodules were arranged in clusters in a near-linear pattern, with a verrucous texture and prominent white hyperkeratosis (Figure 1). Standalone lesions measured up to 1.5 cm in diameter, with a cluster on the right upper extremity measuring 3 x 5 cm. Lesions were confined to the distal extremities, with no involvement of other areas, including mucosal surfaces, hair, or nails. No palpable lymphadenopathy was appreciated, and neurological examination was unremarkable, with intact muscle strength and sensation.

**Figure 2:**
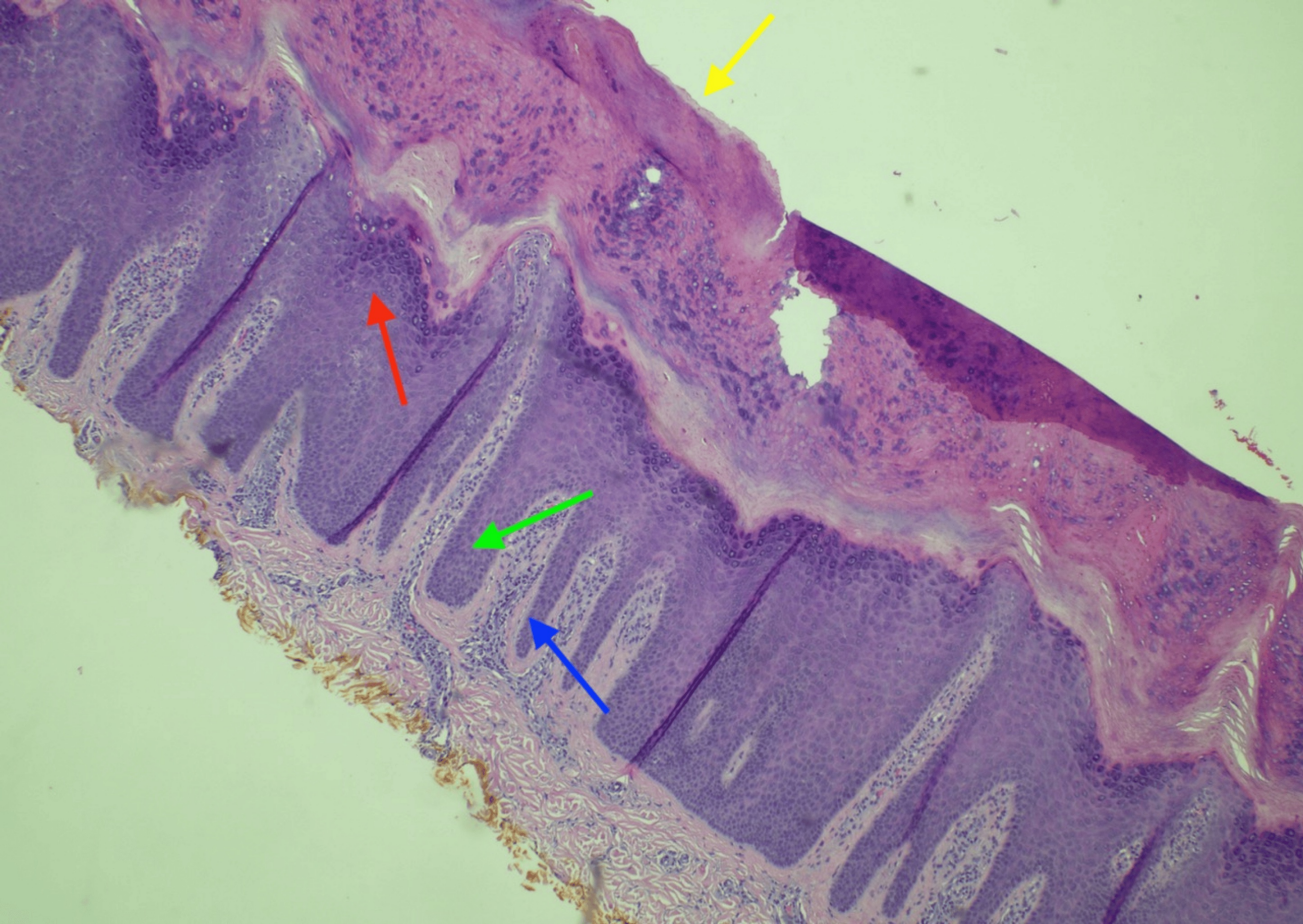
Periodic acid-Schiff (PAS) histologic preparation of a nodular sample obtained via shave biopsy shown at 10x magnification displaying hyperkeratosis (yellow arrow), acanthosis (green arrow), and hypergranulosis (red arrow) with underlying thickening of the papillary dermis by coarse collagen bundles arranged in vertical streaks (blue arrow).

Differential diagnoses included pemphigoid nodularis, hypertrophic lichen planus, verruca vulgaris, eruptive xanthomas, and multicentric reticulohistiocytosis. Laboratory results were within normal limits, including complete blood count, basic metabolic panel, liver function tests, C-reactive protein, and erythrocyte sedimentation rate. The diagnosis of prurigo nodularis was confirmed following a biopsy of a lesion from the distal left lower extremity (Figure 2). Previously, the patient had been prescribed topical betamethasone 0.1% and triamcinolone 0.1% but saw minimal to no improvement with either medication. Treatment with dupilumab was recommended; however, the patient was lost to follow-up after relocating, limiting further management.

## Discussion

Prurigo nodularis characteristically presents in middle-aged individuals and is especially prevalent among African American females [2]. The condition has been associated with various comorbidities, including malignancy, HIV, diabetes, atopic dermatitis, and renal and liver dysfunction [1]. Although the exact pathogenesis is not fully understood, emerging evidence suggests a neuroimmune basis involving inflammation, skin barrier disruption, immune modulation, and fibrous nerve changes [2]. Diagnosis is primarily deduced from patient history and physical examination, with symptoms typically including intense pruritus, often accompanied by burning or stinging sensations [2]. The itch may occur sporadically or continuously and worsens with sweating, heat, or irritation from clothing [2].

Prurigo nodularis typically manifests as symmetric, bilateral nodules or plaques on the extensor surfaces [2]. In this case, however, an unusual pattern is observed, with nodules located asymmetrically on the distal extremities. Less commonly, prurigo nodularis may present on the abdomen, upper back, sacrum, or scalp [2]. Lesions often vary in size (from millimeters to centimeters), color (from flesh-toned to brown or black), and quantity [3]. Chronic prurigo can manifest in various morphologies, including papules, nodules, patches, umbilicated shapes, and linear lesions [4]. Among these, the most prevalent is the nodular morphology, characterized by larger dome-shaped nodules exceeding 1 cm. At the same time, the linear type, which features lesions arranged in a linear pattern as observed in this case, is less common [4]. Lesions of different morphologies may also appear simultaneously in the same patient [4]. Notably, the verrucous texture and white hyperkeratosis observed in this patient's prurigo nodules further contribute to the distinctiveness of this case.

Histopathological examination plays an important role in confirming the diagnosis of atypical presentations of prurigo nodularis. Typical findings include thick orthohyperkeratosis, irregular epidermal hyperplasia, and pseudoepitheliomatous hyperplasia, similar to that seen in Figure 2. Histologic examination can also aid in differentiating prurigo nodularis from similar conditions like lichen simplex and hypertrophic lichen planus [3]. Lichen simplex lesions lack pseudoepitheliomatous hyperplasia and nerve fiber thickening, but these findings alone are insufficient to confirm prurigo nodularis [2]. Accurate differentiation requires a correlation between clinical and histological findings. Both hypertrophic lichen planus and prurigo nodularis share features such as epidermal hyperplasia, hypergranulosis, and compact hyperkeratosis, along with vertically arranged collagen fibers and increased fibroblasts and capillaries in the dermis [2]. However, hypertrophic lichen planus is distinguished by basal cell degeneration confined to the tips of rete ridges, without the band-like inflammation typically observed in prurigo nodularis [2]. 

Topical corticosteroids are the first line in treating prurigo nodularis, with additional therapies including antihistamines, oral immunosuppressants, leukotriene inhibitors, and topical emollients [3]. Recently, the monoclonal antibodies dupilumab and nemolizumab have been approved as treatment options, offering promising efficacy with fewer side effects than traditional methods in many cases [5].

## Conclusions

Currently, there are no universally accepted standardized diagnostic criteria for prurigo nodularis, with diagnosis primarily relying on clinical evaluation. This case highlights an atypical presentation of prurigo nodularis, featuring prominent white hyperkeratosis and asymmetric, near-linear distribution on the distal extremities. Although prurigo nodularis generally presents with symmetrical nodules and intense pruritus on extensor surfaces, this case underscores the importance of recognizing diverse and unexpected patterns. Awareness of such presentations helps clinicians expand their differential and prompts biopsy in ambiguous cases to confirm the diagnosis and guide targeted management. Recognizing these unique patterns fosters earlier intervention and optimal care in managing this chronic and often debilitating condition.

## References

[1] Satoh T, Yokozeki H, Murota H (2021). 2020 guidelines for the diagnosis and treatment of prurigo. J Dermatol.

[2] Mullins TB, Sharma P, Riley CA, Syed HA, Sonthalia S (2024). Prurigo nodularis. StatPearls (Internet).

[3] Kwon CD, Khanna R, Williams KA, Kwatra MM, Kwatra SG (2019). Diagnostic workup and evaluation of patients with prurigo nodularis. Medicines (Basel).

[4] Yook HJ, Lee JH (2024). Prurigo nodularis: pathogenesis and the horizon of potential therapeutics. Int J Mol Sci.

[5] Brooks SG, Yosipovitch G (2024). A critical evaluation of nemolizumab for prurigo nodularis. Expert Rev Clin Immunol.

